# T Cell Responses to Influenza Infections in Cattle

**DOI:** 10.3390/v17081116

**Published:** 2025-08-14

**Authors:** Akanksha Hada, Zhengguo Xiao

**Affiliations:** Department of Animal and Avian Sciences, University of Maryland, College Park, MD 20742, USA; hada@umd.edu

**Keywords:** bovine influenza, T cell immunity, CD4+ T cells, CD8+ T cells, γδ T cells, influenza D virus, H5N1 avian influenza, cross-species transmission, zoonotic influenza

## Abstract

Influenza viruses are major threats to global health, with potential to cause widespread disease in both humans and animals. Cattle, once considered resistant, are susceptible hosts for multiple influenza viruses, including influenza A, C and D, while no evidence currently supports infection with influenza B virus. Cattle serve not only as natural reservoirs for influenza D virus but also as emerging spillover hosts for highly pathogenic avian influenza A strains like H5N1. Their role in sustaining viral circulation, facilitating interspecies transmission, and potentially contributing to viral evolution raises significant concerns about future global outbreaks. As host immunity controls viral clearance and spread, understanding how cattle respond to influenza is essential. While most research has focused on antibody-mediated immunity, T cells play indispensable roles in controlling influenza infections by regulating antibody response, clearing infected cells, and providing long-term protection. However, bovine T cell responses to influenza remain poorly characterized. Given that most research has focused on mice and humans, this review outlines current knowledge of bovine T cell responses to influenza viruses in comparison to these well-characterized models. Cross-species comparative studies are essential to identify species-specific immunity, guide cattle vaccine development, and build predictive models to evaluate future pandemic potential.

## 1. Introduction to Influenza Viruses

Influenza viruses are widespread respiratory pathogens that infect a broad range of hosts, including both humans and animals, with varying levels of disease severity. Among livestock, cattle have increasingly emerged as susceptible hosts for multiple types of influenza viruses [1,2,3,4,5,6,7,8], challenging the long-standing perception of their resistance and revealing their potential role in viral maintenance and cross-species transmission [1,9,10,11,12,13,14,15]. Therefore, understanding how cattle respond immunologically, particularly through T cell-mediated mechanisms, is critical for improving disease control strategies in cattle and evaluating their zoonotic risk.

### 1.1. Classification of Influenza Viruses

Influenza viruses are categorized into four types named A, B, C, and D based on their antigenic differences in their internal proteins, such as nucleoprotein (NP) and matrix protein 1 (M1) [16,17]. These internal proteins are highly conserved within each type but differ significantly between types [18,19,20,21,22]. Moreover, they also exhibit distinct genetic and structural characteristics, especially in their RNA genome organization and surface glycoproteins, which further distinguishes influenza viruses into subtypes. Influenza A virus (IAV) and influenza B virus (IBV) each have eight negative-sense RNA segments, while influenza C virus (ICV) and influenza D virus (IDV) contain seven segments [23]. Among them, IAV is the most genetically diverse and is subtyped based on combinations of its two surface glycoproteins, including hemagglutinin (HA) and neuraminidase (NA), with 18 HA and 11 NA subtypes identified, resulting in 198 potential combinations [24,25]. In contrast, IBV shows limited genetic variation compared to IAV and is divided into only two main subtypes on the basis of HA and NA: B/Victoria and B/Yamagata [26,27,28,29,30]. In contrast, ICV and IDV have a single multifunctional surface glycoprotein called hemagglutinin-esterase-fusion (HEF) protein instead of separate HA and NA proteins. ICV is divided on the basis of HEF protein variation into six subtypes, including Taylor/47, Kanagawa/1/76, Yamagata/26/81, Aichi/1/81, Sao Paulo/378/82 and Mississippi/80 [26,31,32]. IDV, the most recently identified type, shares approximately 50% genetic identity with ICV [26,33], and is grouped into five subtypes based on variability in the HEF: D/swine/Oklahoma/1334/2011 (D/OK), D/bovine/Oklahoma/660/2013 (D/660), D/bovine/Yamagata/10710/2016 (D/Yama2016), D/bovine/Yamagata/1/2019 (D/Yama2019) and D/CA2019 [34]. Therefore, influenza viruses are classified based on their antigenic profiles, including differences in internal proteins, as well as their genomic and structural traits, all of which influence their tissue tropism and host range.

### 1.2. Susceptibility of Cattle to Different Influenza Types

Cattle, previously regarded as resistant to influenza viruses [1,9,10,11,12,13,14], are now recognized as susceptible hosts for multiple types of influenza viruses, including those with zoonotic potentials [1,2,3,4,5,6,7,8,35]. Historically, field detections of IAV in cattle were rare, and infections were assumed to be insignificant [1,9,10,11,12,13,14]. However, experimental studies have demonstrated that cattle can indeed support IAV replication. For instance, calves inoculated with Hong Kong-like H3N2 strains exhibited strain-specific outcomes ranging from asymptomatic virus shedding to clinical respiratory illness [1,36]. Additionally, intranasal administration of the swine H1N1 strain A/sw/IL/75 resulted in clinical respiratory disease, virus shedding, transmission to contact calves, and seroconversion within nine days post-infection [37]. These studies, along with others [1,36,37,38,39,40], indicate that cattle are susceptible to IAV, the most genetically diverse influenza virus type with well-established zoonotic potential [41,42]. Additionally, recent unusual outbreaks of highly pathogenic avian influenza (HPAI) A (H5N1), confirmed in 299 dairy herds in the U.S. across 14 states as of 2024, with affected animals exhibiting symptoms such as fever, anorexia, and marked decline in milk production, have raised significant concerns about the susceptibility of cattle to IAV [2,43]. Moreover, IBV is primarily human-specific, with no serological or experimental evidence supporting bovine susceptibility [1]. In contrast, ICV, which typically causes mild respiratory symptoms in children, has recently emerged as a bovine pathogen. First identified in U.S. cattle with respiratory disease in 2016, ICV has since been detected across several U.S. states and Canadian provinces [3,4,5]. A large-scale cohort study examining 599 cattle linked higher ICV loads to increased incidence of bovine respiratory disease and co-infections with other viruses, indicating its pathogenic relevance in cattle [6]. Furthermore, IDV stands out as the most comprehensively studied influenza virus in cattle. First discovered in pigs in 2011, IDV has since been shown to preferentially infect cattle, which serve as its natural reservoir and amplifying host [7]. Despite partial genetic similarity to ICV, IDV exhibits broader host tropism and environmental stability [44,45]. It has been detected in cattle populations across the world, including North America, South America, Europe, Asia, Africa, and Australia, with seroprevalence exceeding 50% in some adult cattle populations [8,46,47,48,49,50]. In humans, a 2011 study found antibodies to IDV in about 1.3% of the general population in the U.S. and Canada [51]. Moreover, occupational exposure significantly increases the risk of infection, with seroprevalence reaching 97% among cattle-exposed individuals such as farmers in Florida [52], highlighting IDV’s zoonotic potential and emerging public health relevance [53,54]. In cattle, IDV plays a critical role in the bovine respiratory disease complex, where it acts both as a primary pathogen and a co-infection facilitator [6,55,56,57]. Given the growing recognition of cattle as hosts and reservoirs for multiple influenza virus types, including zoonotic strains, it is crucial to understand their immune responses.

## 2. Immune Responses to Influenza Viruses

The outcome of influenza virus infection in cattle is determined by the sequential and coordinated actions of innate and adaptive immunity. The innate immune system responds immediately to viral invasion through pattern recognition receptors (PRRs), initiating interferon signaling, inflammation, and recruitment of effector cells, such as neutrophils, macrophages, and natural killer (NK) cells, to contain early viral replication and spread [58,59,60,61,62,63]. This early response not only restricts infection but also provides the inflammatory cues necessary to activate the adaptive immunity [64,65]. In contrast to the rapid and broad action of innate defenses, adaptive immunity is slower but highly specific. It involves B cells that produce virus-neutralizing antibodies, as well as T cells, including CD8+ T cells that eliminate infected cells and CD4+ T cells that assist both B cells and CD8+ T cells [65,66,67,68]. Once activated, these adaptive responses not only clear the infection but also generate long-lived memory cells that protect against reinfection by the same or similar pathogens [69,70,71,72,73,74,75,76]. In cattle, while innate antiviral pathways against influenza seem to be largely conserved [77,78], the full landscape of adaptive immunity, particularly the roles of systemic and mucosal T cell responses, remains incompletely defined. As cattle encounter diverse influenza viruses through respiratory [1,6,7,36,37] and, in some cases, enteric routes [55], understanding how their immune system orchestrates early viral control and long-term protection is critical. The sections below explore the known and emerging mechanisms of bovine immune defense against influenza viruses, with comparisons to corresponding mechanisms in mice and humans, beginning with innate immune responses.

### 2.1. Innate Immune Responses

The innate immune response serves as the first line of defense against influenza virus infections. It employs chemical, physical, and cellular mechanisms to limit viral replication and initiate adaptive immunity. The mucociliary clearance in the respiratory tract provides the first physical barrier upon viral inhalation, with airway mucus trapping viral particles and expelling them via coordinated ciliary movement by ciliated epithelial cells [58,79,80]. These epithelial surfaces are coated with mucins, including MUC5AC, MUC5B, and MUC1, which bear sialic acid residues that serve as decoy receptors for viral binding; however, IAV’s NA can cleave these mucins, undermining this barrier [79]. Moreover, surfactant proteins SP-A/D in the airway lining fluid inhibit IAV and IBV by binding to glycosylated HA on the surface of the virus [63,80,81,82]. If the virus evades these chemical defenses, it confronts cellular innate immune recognition mechanisms in the epithelia.

Cellular innate immune recognition is mediated by PRRs expressed on epithelial and resident immune cells, which detect conserved viral components. These receptors recognize pathogen-associated molecular patterns (PAMPs), such as viral RNA and replication intermediates [83]. Upon viral invasion, PRRs including retinoic acid-inducible gene I (RIG-I)-like receptors (RLRs), Toll-like receptors (TLRs), and the NOD-like receptor NLRP3 are activated by viral components in the respiratory epithelial cells, alveolar macrophages, and other resident immune populations [58,59,60,61,62]. RIG-I detects uncapped 5′-triphosphate viral RNA in the cytoplasm, while TLR3 and TLR7 recognize double-stranded and single-stranded RNA in endosomes, respectively [59,63]. The NOD-like receptor NLRP3 responds to influenza infection-induced signals and cellular stress to form inflammasomes, which are multiprotein complexes that activate inflammatory responses and induce cell death [59,63]. PRR activation drives transcription of factors such as IRF3, IRF7, and NF-κB, leading to production of proinflammatory cytokines, including TNF-α, IFN-γ, IL-1β, IL-18, and IL-6, along with type I (IFN-α/β) and type III (IFN-λ) interferons [58,59,61,62,63,79,84].

Cytokine signaling orchestrates antiviral response by modulating immune cell recruitment, antigen presentation, effector differentiation, and direct inhibition of viral replication. TNF-α enhances chemokine expression such as CCL2 and CCL5 in lung epithelial cells, which recruit immune cells, such as neutrophils, to the site of infection [61,62,65,79,80,85,86]. Neutrophils mediate early viral control through phagocytosis, reactive oxygen species (ROS) generation, and neutrophil extracellular traps (NETs) formation. However, their overaccumulation can cause collateral tissue damage and compromise epithelial integrity [81,87]. Moreover, IFN-γ enhances dendritic cell migration and antigen presentation, promoting T cell activation [64,88,89]. It further promotes Th1 differentiation of activated T cells and drives antibody class switching to IgG2a in B cells within secondary lymphoid organs such as draining lymph nodes [90,91,92,93]. IgG2a is preferentially recognized by Fc receptors on NK cell to facilitate antibody-dependent cellular cytotoxicity [64,65]. Furthermore, secreted interferons act in both autocrine and paracrine fashions to induce hundreds of interferon-stimulated genes (ISGs), which restrict viral replication at various stages [58,60,94]. ISGs such as MX1 (nuclear) and MXA (cytosolic) that are interferon-induced GTPases with antiviral functions inhibit IAV and IBV polymerase activity, IFITM3 prevents viral membrane fusion during entry, and ISG15 conjugates with viral and host proteins to disrupt replication [58,81,84]. While these responses are critical for antiviral defense, excessive PRR activation can trigger cytokine storms and tissue damage, as seen in severe IAV cases like the 1918 H1N1 pandemic in humans [61,62]. Thus, regulation of these cytokine signaling pathways is essential for effective yet balanced viral defense.

Overall, the innate immune response establishes the first line of antiviral defense by detecting influenza viruses through PRRs located in the cytosol and endosomes, initiating signaling cascades that promote the production of interferons and proinflammatory cytokines, as well as activate antiviral effectors, thereby laying the foundation for downstream immune activation.

Although not well characterized, innate immune responses in cattle appear to be conserved compared to those in mice and humans, suggesting that cattle may mount similar responses to influenza virus infection [77]. For instance, RIG-I signaling could be essential for protecting respiratory epithelial cells from influenza virus infection in cattle [95,96]. Additionally, TLR3 and TLR7 are expressed on myeloid dendritic cells, respiratory epithelium, macrophages and TCRγδ+ T cells in cattle [95,97], highlighting their potential functions analogous to those in murine and human counterparts. In IAV-infected dairy cows, histopathological findings show severe inflammation, viral RNA presence, and neutrophilic infiltration in both respiratory and mammary tissues [2], further supporting the presence of conserved innate immune mechanisms. Like in mice and humans, bovine neutrophils generate reactive oxygen species (ROS) and form NETs, suggesting that NETs may contribute to influenza virus clearance in the respiratory tract while also posing a risk of collateral epithelial damage [98]. Moreover, unlike mice and humans, a subset of bovine neutrophils expresses MHC class II, implying a potential role in antigen presentation to CD4+ T cells and in initiating adaptive immunity [99]. Furthermore, IL-10–producing neutrophils in cattle modulate inflammation during parasitic infections and may similarly attenuate IFN-γ- and TNF-α-mediated lung pathology during influenza infection [99]. In vitro studies demonstrate that bovine neutrophils, in the presence of IL-12, enhance expression of CD25, a T cell activation marker, and promote IFN-γ production by naive CD4+ T cells, suggesting that neutrophil–dendritic cell cooperation could drive Th1-skewed antiviral responses in vivo [100]. Therefore, innate immune mechanisms in cattle can be presumed to mostly parallel those in other mammals such as mice and humans, although species-specific differences warrant further investigation.

### 2.2. Adaptive Immune Responses

The adaptive immune response plays a central role in influenza clearance, immune regulation, and long-term immune protection. It is mediated primarily by antigen-specific T and B lymphocytes, which recognize viral components with high specificity through their T cell receptors (TCRs) and B cell receptors (BCRs), respectively. TCRs recognize viral peptides presented on the major histocompatibility complex (MHC) molecules, while BCRs bind directly to native viral antigens. This antigen-specific recognition initiates clonal expansion, differentiation, and memory formation, providing long-term and subtype-cross-reactive immunity against influenza viruses [65,75,76,101,102].

#### 2.2.1. T Cell Responses

T cells are rapidly activated during influenza infection, driving effector responses and memory formation critical for influenza control. In mice, following influenza infection, lung-resident CD103+ dendritic cells capture viral antigens and migrate to the mediastinal lymph nodes via CCR7-CCL19/21 signaling, where they activate naive T cells [65,101,102]. Naive T cells require three critical signals for full activation: (1) antigen recognition through TCR binding to peptide–MHC complexes on antigen-presenting cells (APCs); (2) co-stimulatory signaling, typically via CD28 interaction with B7 molecules (CD80/CD86) on APCs, which enhances and sustains the activation; (3) cytokine signaling, which directs T cell differentiation, proliferation, and survival [103]. Upon activation, T cells upregulate tissue-homing receptors such as CXCR3, which guide their migration towards CXCL9/10 chemokine gradients produced by alveolar macrophages and epithelial cells [67,104]. Within the infected lungs, CD4+ T cells secrete IFN-γ to suppress viral replication and assist B cells in antibody class-switching via CD40L interactions [65,66,67,68], whereas CD8+ T cells mediate cytotoxic activity through perforin/granzyme B and FasL/TRAIL pathways, as well as secrete effector cytokines such as IFN-γ and TNF-α that shape the antiviral environment [105,106,107,108]. Their optimal activation is supported by CD4+ T cell help, particularly through CD40-CD40L interaction that license APCs, and through the production of cytokines such as IL-2, which enhance the magnitude and longevity of CD8+ T cell responses [109]. Both CD4+ and CD8+ T cells generate memory subsets, including central memory (TCM), effector memory (TEM), and tissue-resident memory (TRM) populations, which enable rapid and localized responses upon reinfection [69,70,72,73,110,111]. In cattle, although detailed mechanistic studies remain limited, emerging evidence supports the presence of robust T cell responses to both IAV and IDV, with virus-specific CD4+ and CD8+ T cells detected in respiratory and mammary tissues following infection or vaccination [2,7,112,113,114]. These T cell populations are likely essential for cross-protective immunity across influenza subsets and for long-term viral control in cattle as in mice and humans.

##### CD4+ T Cell Responses

CD4+ T cells mount antiviral responses against influenza by facilitating humoral immunity, supporting CD8+ T cell priming and mediating immune regulation. These cells are activated upon recognizing influenza epitopes, such as the HA stalk (residues 113–131), NA catalytic domain, and M2 ectodomain, presented on MHC class II molecules through their TCRs [115,116,117,118,119]. Upon activation, CD4+ T cells differentiate into functional subsets, including T follicular helper (Tfh), Th1, Th2, Th17, and regulatory T cells (Tregs), in response to cytokine cues, with each subset performing specialized immune functions. Memory CD4+ T cells, generated following primary infection, provide long-lasting immunity by quickly responding to re-exposure to influenza, ensuring rapid and robust immune protection.

Tfh and Th1 cells mediate both humoral and cellular immunity. Tfh help in antibody-mediated protection against influenza virus. IL-6 signaling favors Tfh lineage differentiation of CD4+ T cells by suppressing IL-2 responsiveness through inhibition of CD122 upregulation [120]. Tfh cells support germinal center (GC) formation and B cell affinity maturation through IL-21 and CD40L interactions [104,115,116,117,121]. These cells require SAP-dependent interactions with B cells through SLAM family receptors, such as Ly108 and CD84, to maintain productive T cell–B cell interactions [122]. Through these mechanisms, Tfh cells sustain GC reactions and promote IL-21 secretion for high-affinity IgG antibody production by B cells in response to influenza virus [123,124,125]. Moreover, IFN-γ produced by Th1 cells influences antibody isotype switching in B cells, promoting class switching to IgG2a in mice, an isotype associated with effective antiviral functions such as opsonization and complement activation during influenza infection [126,127]. Additionally, Th1 cells reinforce antiviral defenses by licensing dendritic cells and amplifying CD8+ T cell cytotoxicity. Th1 differentiation is driven by IL-12/STAT4 signaling from dendritic cells in the mediastinal lymph nodes, leading to T-bet and IFN-γ expression in response to influenza infection [121,128,129,130]. Th1 cells secrete IL-2 to sustain T cell proliferation in lymph nodes while directly suppressing viral replication in lung tissue via IFN-γ production [121,128,131]. Furthermore, Th1-derived IL-2 and IFN-γ enhance CD8+ T cell cytotoxic responses [104,115,116,117,121]. Therefore, Tfh and Th1 subsets drive effective humoral and cellular immune responses that provide protective immunity against influenza virus infection. However, the overall immune response must be tightly regulated to prevent excessive inflammation and immunopathology. CD4+ T cells regulate the magnitude and quality of the immune response to limit immunopathology. Th2 responses, induced by IL-4, often impair antiviral immunity by antagonizing Th1 responses and suppressing CD8+ T cell activation [132,133]. Th17 cells, emerging later in infection, secrete IL-17A/F and IL-22 that exhibit dual functionality. For instance, IL-17 recruits neutrophils and synergizes with TNF-α/IL-1β to exacerbate lung pathology in severe IAV infections [65,134,135]. However, IL-22 promotes epithelial repair, counterbalancing IL-17-mediated damage [65,135]. TGF-β signaling polarize CD4+ T cells into Tregs during influenza infection [136]. These Tregs stably express FoxP3 and prevents virus-induced immunopathology by mediating tissue protection rather than viral clearance [137]. The reduced inflammatory damage is achieved by the production of IL-10 and TGF-β by Tregs, which suppress T cell proliferation in lungs and inhibits NETs formation [65,121,138]. Overall, CD4+ T cells contribute to antiviral defense by orchestrating antibody responses, enhancing CD8+ T cell activation, and regulating immune balance.

Memory CD4+ T cells play a crucial role in long-term immunity against influenza by supporting both localized and systemic responses upon re-exposure. These memory populations include tissue-resident memory T cells (TRMs) that permanently occupy infection sites, as well as circulating central memory (TCMs) and effector memory (TEMs) cells that patrol lymphoid and peripheral compartments, such as the lungs [115]. TCMs express lymphoid-homing receptors such as CD62L and CCR7, and primarily reside in secondary lymphoid organs, whereas TEMs lack these receptors and migrate through peripheral tissues [139,140]. Following influenza infection, virus-specific TEMs predominate in peripheral sites, whereas TCMs are enriched in lymph nodes. TRMs, in contrast, express low levels of CD62L and CCR7 like TEMs but are distinguished by high expression of tissue-retention markers such as CD69 and, to a lesser extent, CD103, which facilitate their long-term residence within lung tissue [115]. After influenza infection, chemokines such as CXCR6, direct a subset of effector CD4+ T cells to the lung airways and parenchyma, where they upregulate CD69 and CD103 to establish long-lived TRMs [69,70,115,131,141]. Upon re-exposure to influenza antigens, these memory subsets promptly recognize viral peptides presented by local APCs and produce cytokines such as IFN-γ, TNF-α, and IL-2 within hours [115,131]. In mice, adoptive transfer of influenza-specific in vitro-generated memory CD4+ T cells into naive hosts induces a rapid upregulation of pulmonary and systemic inflammatory cytokines, including IFN-γ, IL-12, IL-1, and IL-6, as well as chemokines, such as CXCL9, CXCL10, and CCL2, within 48 h of heterosubtypic influenza challenge. This early cytokine response correlates with reduced viral titers and improved survival [141,142]. While influenza viruses primarily target the respiratory tract, emerging evidence of IDV being present in the intestine suggests possible role of intestinal TRMs or intraepithelial T lymphocytes (T-IELs) in mucosal defense [55,143,144]. In mice, lung-derived CCR9+CD4+ T cells migrate to the small intestine via the CCL25/CCR9 axis during influenza infection, contributing to dysbiosis and IL-15-driven Th17 polarization that exacerbates IL-17A-mediated intestinal injury [71]. Although direct evidence of intestinal TRMs and T-IELs in influenza is limited, their established roles in maintaining epithelial integrity and immune surveillance in humans, mice, and cattle suggest their contribution to host protection during systemic or enteric influenza exposure [55,143,144]. Altogether, TRMs, primarily in the lungs and potentially in the intestine, along with TEMs and TCMs, contribute to rapid immune responses that limit viral replication, preserve tissue integrity, and enhance protection at both systemic and local sites upon reinfection with influenza viruses.

Although bovine research has increasingly focused on IAV and IDV in recent years [2,112], our understanding of bovine CD4+ T cell responses to influenza viruses remains limited. Nevertheless, CD4+ T cells in cattle are expected to exhibit conserved antiviral functions across different influenza infections, while also showing virus-specific adaptations in epitope recognition, cross-reactivity, and regulatory mechanisms. These responses are likely influenced by host MHC class II diversity and viral antigen conservation. For instance, CD4+ T cells recognizing highly conserved influenza proteins can generate cross-protective immunity across different IAV subtypes in cattle [113]. These antigen-specific CD4+ T cells play a key role in controlling and preventing influenza virus infection in the bovine mammary gland [113]. Moreover, recent preprint reported that a mRNA-lipid nanoparticle (mRNA-LNP) vaccine targeting H5 HA protein of HPAI IAV induces proliferation of peripheral CD4+, CD8+ and TCRγδ+ T cells that produced IFN-γ in Holstein calves [114,145]. IFN-γ is known to promote IgG2 class-switching in cattle [146,147,148], suggesting analogous helper functions during influenza virus infection. Furthermore, IDV infection induces mild upregulation of both T-bet and Gata-3 transcription factors at later stages, suggesting mixed Th1/Th2 lymphocyte activation [8]. This Th1/Th2 mixed phenotype has been detected in response to gastrointestinal parasite in cattle [149], indicating the difference in bovine CD4+ T cell response as compared to in mice and humans. Most evidence comes from serological studies, viral detection, and clinical observations rather than detailed immunological analysis of CD4+ T cell responses [1]. However, despite the growing recognition of bovine susceptibility to influenza viruses, direct characterization of CD4+ T cell responses in cattle remains extremely limited.

##### CD8+ T Cell Responses

CD8+ T cells are central to antiviral defense against influenza, playing a key role in clearing infected cells through cytotoxic activity and contributing to long-term immunity. These cells share several functional features with CD4+ T cells, including cytokine production, memory formation, and support for coordinated immune responses, but are uniquely specialized for direct elimination of virus-infected cells.

CD8+ T cells execute cytotoxic functions that directly eliminate infected cells and shape the inflammatory microenvironment during influenza virus infection. These cells are activated upon TCR recognition of influenza viral proteins presented via MHC-I on infected cells or cross-presented by dendritic cells in draining lymph nodes, followed by co-stimulatory signaling through CD28 interaction with B7 molecules (CD80/CD86) and cytokine signaling that directs T cell differentiation, proliferation, and survival [103,105,106]. Although antigen-independent “bystander” CD8+ T cell activation occurs, antigen-specific responses remain dominant [73,150]. For instance, during acute influenza infection, lung CD8+ T cells lack CD25 expression and proliferate nonspecifically, driven by inflammatory cytokines like IL-15 but plays a limited role in viral clearance [73,150]. Antigen-specific CD8+ T cells upregulate lung-homing receptors such as CXCR3 and infiltrate infected tissues [106,107], where they undergo clonal expansion and eliminate infected respiratory epithelial cells through perforin/granzyme B-mediated cytotoxicity and apoptosis-inducing pathways involving FasL and TRAIL [107,108]. The magnitude of this response correlates with antigen load, i.e., higher viral doses induce larger CD8+ T cell populations with enhanced cytokine production and cytotoxicity [105,151]. Moreover, sustained IL-12 signaling from surrounding cells drive T-bet-dependent Th1 polarization and effector differentiation of activated CD8+ T cells, secreting IFN-γ and TNF-α, further amplifying innate immunity by recruiting neutrophils and inflammatory dendritic cells [105,106,152,153]. CD8+ T cells targeting conserved viral internal proteins, such as nucleoprotein (NP) and polymerase basic protein 1 (PB1), exhibit cross-reactivity and protection across influenza strains and subtypes [151,154,155,156]. Overall, CD8+ T cells drive viral clearance and cross-strain protection through cytotoxicity and cytokine-mediated immune responses.

Memory CD8+ T cells, including circulating memory and lung-resident TRM, contribute to rapid and durable protection during reinfection [72,73]. These memory CD8+ T cells persist in both circulating and tissue-resident compartments, each with distinct protective roles during secondary influenza challenge. Circulating memory CD8+ T cells are categorized as central memory TCM (CCR7+CD62L+) or effector memory TEM (CCR7−CD62L−). TCM patrol secondary lymphoid organs, while TEMs surveil non-lymphoid tissues. During influenza challenge, TEMs rapidly migrate to the lungs, where they proliferate and acquire cytotoxic function [72,73]. Meanwhile, TRMs constitutively reside in the lung epithelium, which is marked by CD69 and CD103 expression [110,111]. These cells are primed for immediate effector function, producing IFN-γ within hours of viral re-encounter [74]. Unlike peripheral TEM, TRM cells exhibit low basal granzyme B and perforin levels to minimize epithelial damage but upregulate these molecules upon re-infection [74]. Furthermore, CD8+ T cells targeting conserved internal proteins such as NP, M1, and PB1 exhibit cross-reactivity across different influenza types, enabling TRMs to recognize these heterotypic strains [111,154,156]. Cross-reactive CD8+ T cells are enriched in peripheral blood and lungs, with CD38+Ki67+ effector populations detectable during active influenza infection in mice [154,157]. Nevertheless, multi-epitope vaccines targeting conserved regions of NP and PB1 synergistically enhance cross-protection in IAV subtypes, reducing viral titers by 90% in murine models [151,156]. Therefore, while TRMs in the lungs provide frontline defense, peripheral circulating CD8+ T cells, including TCM and TEM subsets, play important roles in systemic surveillance and secondary responses, mediating viral clearance through cytotoxic mechanisms.

In cattle, emerging evidence suggests that conserved CD8+ T cell mechanisms contribute significantly to influenza control, particularly against IDV and IAV strains. Intranasal IDV challenge led to peak viral replication in nasal turbinates and tracheal tissues, associated with robust CD8+ TRM generation and reduced viral shedding in cattle [7]. Moreover, during recent H5N1 clade 2.3.4.4b IAV outbreaks in dairy herds, intra-mammary infection elicited virus-specific CD8+ T cell responses in mammary lymph nodes, with IFN-γ-producing CD8+ T cells contributing to viral clearance from milk within 21 days [2,113]. Additionally, a preprint study observed a strong expansion of antigen-responsive CD8+ T cells producing IFN-γ in cattle vaccinated with H5 mRNA-LNP formula [114,145]. Together, these findings suggest that although underexplored, CD8+ T cell immunity in cattle is functionally robust and likely critical for limiting both respiratory and systemic influenza virus infections.

##### B Cell Responses

With the help from CD4+ T cells, B cells contribute to influenza immunity by producing antibodies that mediate viral neutralization, opsonization, phagocytosis, complement activation, and antibody-dependent cellular cytotoxicity, while also generating immunological memory and adapting to antigenic variation across influenza subtypes [158,159,160].

Antibodies produced by B cells mediate protection against influenza through multiple effector mechanisms. Neutralizing antibodies bind to viral surface proteins, such as HA, NA, or HEF, and block viral entry into host cells, preventing infection at an early stage [45,161,162]. This function, particularly mediated by IgG1 and IgG2a isotypes in mice, serves as a primary indicator of vaccine-induced protection [161,163,164]. In addition to neutralization, antibodies facilitate opsonization by coating viral particles or infected cells and enhancing their uptake by phagocytes via Fc receptors, a process known as antibody-dependent cellular phagocytosis [165,166]. Phagocytosis enables monocytes, macrophages, and dendritic cells to degrade viral particles and present viral antigens to T cells [162,165,166]. Complement activation also contributes to viral clearance. Antigen–antibody complexes initiate the classical complement pathway, leading to membrane attack complex formation on virus-infected cells and enhanced opsonization [167,168,169]. Antibody-dependent cellular cytotoxicity, primarily mediated by NK cells via FcγRIII (CD16) engagement, allows for targeted killing of infected cells coated with antibodies [89,170,171]. Lysis of infected cells can release viral particles into the interstitial fluid, where they become exposed to circulating neutralizing antibodies that can immediately bind and neutralize them, preventing further spread [168,172,173]. Together, these antibody-mediated mechanisms function synergistically to contain and clear influenza virus infection.

Although limited T cell-independent responses that are driven by type I interferons (IFN-I) and Toll-like receptor (TLR) engagement have been observed, B cell activation is primarily coordinated through CD4+ T cell-dependent pathway. IFN-I upregulates B cell activation markers, such as CD69 and CD86, and promote their differentiation into antibody-secreting cells within 48 h of infection [169,174,175]. TLR signals, particularly through TLR3 and TLR7, further support robust B cell responses [175]. However, T cell-dependent B cell responses are essential for sustained and high-affinity immunity. These responses require cognate interactions with CD4+ T cells, particularly Tfh cells, which drive germinal center (GC) formation, class-switch recombination, and affinity maturation through CD40-CD40L interactions and IL-21 signaling [75,76]. Although some long-lived plasma cells (LLPCs) may arise from extrafollicular responses outside of GCs, lower-affinity B cell clones are predominantly recruited into GCs, where they undergo somatic hypermutation to differentiate into LLPCs [75,76,169,175,176,177,178]. High-affinity responses are predominantly shaped within GCs, which typically peak 2–3 weeks post-infection and persist for weeks following viral clearance [75,76]. For instance, pre-existing memory B cells, generated through natural infection or vaccination, dominate recall responses to antigenically novel IAV and IBV strains [179,180,181]. The breadth of reactivity is shaped in part by immune imprinting during early-life exposures, which biases the immune system toward specific viral epitopes encountered during initial infections [180,182]. Recognition of conserved regions like the HA stalk facilitates cross-reactivity across influenza types and subtypes. For instance, in IAV, certain memory B cell lineages can neutralize both highly pathogenic group 1 subtypes such as H1 and H5, as well as low-pathogenic group 2 subtypes like H3 and H7 [169,179]. In contrast, B cell responses to IBV are more limited between the Victoria and Yamagata subtypes due to substantial antigenic divergence in the HA head region, which reduces cross-reactivity [29,30]. For ICV and IDV, the HEF protein serves as the primary antigenic target. Despite antigenic drift, a gradual and continuous accumulation of antigen mutations, HEF retains conserved receptor-binding and fusion domains that are capable of inducing broadly reactive antibody responses [55,183]. B cell responses to influenza are predominantly CD4+ T cell-dependent, with T follicular helper cells playing a central role in driving high-affinity antibody production.

Although information on bovine B cell responses to influenza viruses remains limited, current evidence indicate that B cells employ conserved defense mechanisms across different influenza types. For instance, cattle mount robust B cell responses against IAV subtypes such as H1N1, H5N1, and H3N2, as well as ICV and IDV, but not against IBV, as evidenced by serosurveillance studies in bovine populations [1,6,184]. In the case of IAV, virus-neutralizing antibodies are detectable in bovine serum by seven days post-infection and in milk by 14 days following H5N1 exposure [2]. Moreover, a preprint has reported that mRNA-LNP vaccination against H5 protein triggers strong IgG antibody responses against both the vaccine strain (A/Astrakhan/3212/2020) and the cattle-derived H5N1 virus [114,145]. Seroprevalence studies in Africa indicate widespread exposure of cattle to both ICV and IDV [184]. ICV induces antibodies targeting conserved regions of its HEF peptide and receptor-binding domain, allowing cross-reactivity [3,184]. For IDV, the HEF protein is the primary B cell target. DNA vaccines expressing a consensus HEF sequence can induce neutralizing antibodies that prevent infection in experimental models [23,34,185]. However, antigenic variation between major IDV subtypes, such as D/OK and D/660, poses challenges for cross-protection. Antibodies generated against one subtype often show reduced efficacy against another due to subtype-specific epitopes located in the HEF apex [34,185]. Furthermore, the CD4+ T cell-dependent CD40-activated B cells in cattle skew toward IgG2 production for enhanced opsonization and clearance of viral particles [95,146,186,187]. Together, these findings suggest that cattle generate functional and partially cross-reactive humoral responses that is shaped by conserved viral epitopes.

### 2.3. Balance Between Viral Clearance and Immunopathology in Influenza-Specific T Cell Responses

Effective T cell-mediated immunity against influenza requires a delicate balance between viral clearance and limiting immunopathology, as most clinical syndromes and deaths are driven by excessive inflammation and tissue damage despite successful control of infection [188,189].

While essential for antiviral defense, influenza-specific CD8+ and CD4+ T cells can contribute to lung injury through proinflammatory cytokine production and epithelial damage. Adoptive transfer studies demonstrate that CD8+ T cells can induce severe lung pathology by producing IFN-γ and TNF-α, which drive chemokine-mediated infiltration of myeloid cells and promote apoptosis of alveolar epithelial cells [190,191]. IFN-γ enhances CD8+ T cell cytotoxicity and viral clearance but simultaneously suppresses the IL-6/STAT3 axis critical for epithelial repair, exacerbating tissue injury [192]. Human studies mirror this complexity, with elevated IFN-γ levels in severe influenza cases correlating with both immunopathology and survival, suggesting dose- and timing-dependent effects [192]. TNF-α blockade reduces lung injury but compromises CD8+ T cell recruitment, while human data associate high TNF-α levels with both severe disease and recovery, suggesting context-specific roles in influenza infections [191]. Moreover, the dual role of T cells in influenza infection, mediating viral clearance while risking immunopathology, is further demonstrated by studies examining the effects of IL-15 signaling and CD8+ T cell activity. For instance, IL-15 knockout mice exhibit reduced mortality despite unchanged viral titers as a result of diminished CD8+ T cell-mediated lung injury rather than impaired influenza control [193]. IL-15 promotes CD8+ T cell migration to the lung via chemotactic signaling and enhances their survival, amplifying both effector responses and immunopathological potential [194]. Therapeutic administration of IL-15 complexes during infection expands lung-resident CD8+ T cell memory pools, thereby improving cross-protective immunity. However, these effects also indicate the potential of IL-15 to exacerbate inflammatory responses when dysregulated [194]. These findings highlight IL-15’s dual role as a facilitator of protective memory and a contributor to acute injury. Furthermore, IL-17 produced by Th17 cells promotes neutrophil-driven lung and intestinal injury during influenza, yet IL-17A is also essential for mucosal repair in neonates, revealing age- and context-dependent roles [71,135,195]. These findings underscore that the pathogenic outcomes of T cell responses are highly dependent on cytokine context, dose, and host factors such as developmental stage and tissue environment. However, immune system must also employ regulatory mechanisms to modulate excessive inflammation and tissue damage.

Tregs are critical for restraining effector T cell activity and limiting tissue damage during influenza infection. Tregs limit CD8+ T cell and NK cell proliferation via IL-10 and TGF-β production while promoting Tfh cell differentiation in CD4+ T cells by sequestering IL-2, thereby enhancing germinal center B cell responses in influenza infections [196]. Treg depletion exacerbates lung injury due to unchecked effector T cell activity and impairs antibody maturation, illustrating their regulatory functions [196]. Tregs also mitigate post-clearance pathology, as their inhibition during recovery prolongs neutrophilic inflammation and delays tissue repair [193,196]. Inhibitory receptors such as PD-1 and Tim-3 also play regulatory roles; however, excessive signaling through these pathways may lead to T cell exhaustion and reduced viral control [190]. Therefore, Tregs and inhibitory pathways serve as key modulators of immune balance during influenza, preventing immunopathology while preserving effective antiviral responses.

Together, these findings underscore the context-dependent roles of T cells and cytokines in influenza, where protective immunity is intricately linked to the risk of immunopathology, emphasizing the need for finely tuned immune regulation to achieve optimal outcomes. While these mechanisms have been primarily characterized in mice and humans, similar regulatory mechanisms likely operate in cattle, where T cell-mediated responses must also balance effective viral clearance with the preservation of tissue integrity. Further research is needed to understand these processes in bovines.

### 2.4. Comparison of Peripheral T Cell Responses in Human, Mice, and Cattle

T cell responses to influenza viruses might differ markedly between species, with mice and humans relying primarily on MHC-restricted TCRαβ+ T cells, while cattle exhibit a TCRγδ+ T cell-dominant profile in both circulation and mucosal sites that suggests distinct, evolutionarily adapted mechanisms of antiviral defense. Human and murine T cell responses to IAV and IBV are dominated by TCRαβ+CD8+ and TCRαβ+CD4+ T cells targeting conserved internal proteins like NP and M1. These responses are shaped by MHC-I/II presentation, with TCRαβ clonotypes driving cross-protective memory in mice and humans [197,198]. While less well-characterized, bovine TCRαβ+ T cell responses to influenza viruses is likely similar to those in mice and humans, with TCRαβ+CD8+ and TCRαβ+CD4+ subsets recognizing viral proteins through MHC-restricted mechanisms [1,2,7,112,113,114]. In contrast, cattle, apart from using TCRαβ+ T cells response, may also employ TCRγδ+ T cells as one of the first responders due to their abundance in both circulation and mucosal sites [143,185,199,200,201,202], early tissue recruitment during infections [203,204], and ability to respond to inflammatory cytokines and pathogen-associated molecular patterns (PAMPs) [205,206]. Cattle exhibit a starkly different T cell landscape where TCRγδ+ T cells constitute 60–70% of peripheral blood lymphocytes in calves and ~30% in adults [185,199,200]. These cells are highly functional, as in bovine mycobacterial infection models, TCRγδ+ T cells are rapidly recruited to nascent granulomas, with distinct subsets accumulating within days of *Mycobacterium* challenge [203,204]. WC1+ TCRγδ+ T cells in neonatal calves rapidly secrete large amounts of IFN-γ within hours of stimulation by innate cytokines such as IL-12 and IL-18, independent of TCR engagement or antigen-presenting cell involvement [205]. Following BCG vaccination, bovine TCRγδ+ T cells exhibit features of innate immunity, where upon in vitro re-exposure to bacterial PAMPs such as *E. coli* LPS or Pam3CSK4, they mount a rapid inflammatory response characterized by IL-6 and TNF-α production [206]. Bovine TCRγδ+ T cells also express TLR3 [206], which may recognize influenza virus-derived double-stranded RNA intermediates within endosomal compartments, thereby initiating downstream antiviral responses. Moreover, in mice and humans, TCRγδ+ T cells produce IL-17A early during IAV infection, promoting neutrophil recruitment and epithelial repair [207,208,209]. This mechanism may be even more pronounced in cattle. Furthermore, bovine TCRγδ+ T cells represent a major immunoregulatory T cell subset that produce IL-10 and inhibit the proliferation of activated CD4+ and CD8+ T cells in vitro [143,210]. These features of bovine TCRγδ+ T cells highlight their importance in viral immunity and tissue repair. While in mice and humans, TCRαβ+CD8+ T cells can contribute to immunopathology through excessive TNF-α and IFN-γ production, cattle’s abundant mucosal TCRγδ+ T cells may mitigate such damage by promoting IL-22-mediated epithelial repair, aligning with the generally low pathogenicity of IAV and IDV in its natural bovine host [1,9,10,11,12,13,14,36,37,38,39,40,143,185,199,210]. These interspecies differences highlight the unique immunological mechanisms of cattle where, although bovine TCRαβ+ T cell responses to influenza viruses appear broadly similar to those in mice and humans, TCRγδ+ T cell responses may play a more prominent role in early antiviral defense, providing rapid, MHC-independent protection against influenza viruses.

### 2.5. Immune Evasion by Influenza Viruses

Influenza viruses have evolved sophisticated mechanisms to evade host immunity, driving their continued persistence and spread. This evasion primarily occurs through two distinct evolutionary processes affecting surface proteins, antigenic drift and antigenic shift, as well as through the expression of viral proteins that antagonize host immune responses.

Antigenic variation helps influenza viruses evade host immunity to ensure their persistence and spread. Antigenic drift represents the gradual, continuous accumulation of mutations in the genes encoding the major surface glycoproteins HA and NA [85]. For HA, two main molecular mechanisms underlie antigenic drift, which are amino acid substitutions that alter the biophysical properties of antigenic epitopes, reducing antibody binding, and the introduction of N-linked glycosylation sites in the HA globular head domain, which sterically shield antigenic sites from neutralizing antibodies [211]. Retrospective analyses have shown that head glycans are added to H1 and H3 HAs in IAVs at regular 5- to 7-year intervals until reaching an empirically defined glycan limit, after which glycans swap positions rather than being added [211]. While the HA stalk region has been considered a target for broadly neutralizing antibodies, recent evidence suggests it may also be subject to antigenic drift. In vitro experiments have identified mutations conferring escape from broadly neutralizing stalk antibodies [211]. Moreover, NA also undergoes antigenic drift, albeit asynchronously to HA [211]. Recent studies have identified mutations near the enzyme active site of a NA subtype of IAV, N2, that escape monoclonal antibodies and are under positive selection [211]. The fixation of an N-linked glycosylation site in N2 at position 245 and P468H resulted in substantial antigenic drift of N2, with the attached glycan sterically shielding NA-inhibiting antibodies [211]. In contrast, antigenic shift represents a mechanism by which influenza evade host immunity through abrupt, major changes in surface proteins HA and/or NA via genetic reassortment of gene segments between different viral strains [85]. This process occurs when two or more different influenza viruses, specifically IAV, co-infect the same host cell, allowing for the exchange of entire gene segments among the eight-segmented viral genome [85,212]. Unlike the gradual mutations of antigenic drift, antigenic shift produces viruses with surface proteins that are antigenically distinct from previously circulating strains, often reducing the effectiveness of pre-existing immunity and facilitating more efficient viral transmission [85,212]. Both antigenic drift and shift undermine host defenses, allowing influenza viruses to evade immune detection and maintain their infectious cycles.

Beyond antigenic evolution, influenza viruses encode proteins that directly antagonize host innate immune responses. For instance, IAV Non-Structural Protein 1 (NS1) protein inhibits RIG-I-mediated interferon responses by binding TRIM25, preventing RIG-I ubiquitination and downstream IFN induction [213,214]. This interaction is conserved across human, avian, and swine strains but absent in mice [214]. NS1 also induces apoptosis in human airway epithelial cells via a caspase-dependent mechanism during IAV infection [215]. In contrast, IBV NS1 blocks RIG-I activation by binding the 5′-triphosphate ends of viral RNA, without interacting with TRIM25 [216]. The NS1 proteins of IAV and IBV impair dendritic cell maturation by downregulating costimulatory molecules CD80/86 and IL-12 expression, thereby compromising their function [217]. This leads to suppressed Th1 polarization, as NS1-expressing viruses induce lower IFN-γ production in T cell cocultures [217]. NS1 from ICV and IDV suppresses RIG-I signaling via its C-terminal domain in a TRIM25-independent manner, limiting IFN-β promoter activity despite modest overall IFN antagonism [218,219,220], possibly explaining their mild pathogenesis. Additionally, IAV NA can cleave latent TGFβ into its active form, further helping virus limit the inflammatory response [58]. Moreover, the RNA-dependent RNA polymerase components and Matrix Protein 1 (M1) of IAV have demonstrated antagonism of IFN responses [220]. Furthermore, IDV M1 bypasses RIG-I altogether by promoting TRAF6 degradation via KEAP1-mediated, K48-linked ubiquitination, thereby blocking NF-κB and IFN-β signaling [221]. The IAV accessory proteins, such as polymerase basic protein 1-Frame 2 (PB1-F2) and Polymerase Acidic Protein-X (PA-X), encoded through alternate reading frames or frameshifts of polymerase components, further enhance immune evasion. PB1-F2, especially with N66S mutation, binds mitochondrial antiviral signaling protein and inhibits the initiation of IFNs. The 1918 deadly IAV strain H1N1 and H5N1 with N66S mutation enhanced viral replication in the lung [222]. PB1-F2 acts as a virulence factor, increasing cell death, reducing viral clearance, and enhancing viral gene expression [220]. PA-X decreases type I IFN expression by preventing innate immune receptor signaling and facilitates degradation of host mRNAs [223,224,225,226]. Moreover, IAV have evolved other immune evasion strategies, such as NA-mediated removal of sialic acid residues from NK cell receptors like NKp46, impairing their ability to recognize HA on infected cells [64,227,228]. Collectively, these viral immune evasion proteins modulate host innate sensing, disrupt interferon signaling pathways, impair antigen-presenting cell function, and inhibit effector lymphocyte activation, enabling immune evasion and enhancing viral fitness within the host.

Another critical immune evasion strategy employed by influenza viruses involves MHC regulation. IAV and IBV can downregulate MHC-I surface expression to evade CD8+ T cell recognition. IAV degrades MHC-I globally via proteasomal pathways, while IBV traps MHC-I in the Golgi apparatus, reducing peptide presentation [155]. This immune evasion is temporal, peaking during late infection when viral egress is maximal [155]. Consequently, CD8+ T cells exhibit delayed recognition of infected cells, allowing transient viral persistence [155]. Beside these mechanisms, IDV exhibits unique environmental resilience demonstrated by its ability to remain stable at pH 3.0 and temperatures up to 53 °C due to their HEF structure [229]. These features may contribute to its persistence and transmission within their hosts. By downregulating antigen presentation and enhancing environmental stability, influenza viruses prolong infectivity and hinder immune-mediated clearance.

In cattle, the recent HPAI H5N1 Clade 2.3.4.4b IAV infection in dairy cattle highlights adaptive mechanisms for immune evasion. The Texas H5N1 isolate with B3.13 genotype demonstrates enhanced replication in bovine lung cells and antagonizes RIG-I/MDA5 signaling pathways, suppressing IFN-β production [230]. NS1 proteins in bovine-adapted H5N1 strains counteract IFN responses by targeting host factors like RIG-I and MDA5, while NP mutations allow escape from MX1 restriction, reflecting mammalian-specific adaptation [230,231]. Moreover, IDV in cattle employs the M1 to suppress RIG-I-TRAF6 signaling by recruiting the E3 ubiquitin ligase KEAP1, which promotes K48-linked polyubiquitination and proteasomal degradation of TRAF6, effectively blocking NF-κB and IFN-β induction [96,232]. In addition, IDV has been shown to infect the bovine intestinal tract, likely facilitated by its exceptional acid and thermal stability [229], allowing replication under harsh physiological conditions.

Overall, influenza viruses employ diverse immune evasion strategies, including antigenic drift and shift, suppression of host innate responses, and MHC-I downregulation, which are predominantly characterized in human and murine models. Although emerging data from bovine-adapted IAV and endemic IDV strains suggest that similar mechanisms may operate in cattle, such as NS1- and M1-mediated IFN antagonism, the molecular details remain poorly defined.

### 2.6. Influenza Vaccines

The efficacy of influenza vaccines across species is shaped by the vaccine platform and delivery route, both of which influence the quality, localization, and longevity of T cell-mediated immune responses. Live attenuated influenza vaccines (LAIVs) consistently induce robust TRMs in respiratory mucosa, with intranasal LAIV administration generating lung CD8+ TRMs in ferrets and pigs that persist for more than 3 months and mediate cross-strain protection independent of circulating antibodies [233]. This contrasts with inactivated influenza vaccines (IIVs), which primarily stimulate neutralizing antibodies but fail to establish TRM populations [233,234]. In children, LAIV elicits multifunctional Th1 cells (IFN-γ+/IL-2+/TNF-α+) that persist more than one year, correlating with reduced symptomatic infection despite low serum Hemagglutination inhibition titers [235,236,237], while adults exhibit diminished T cell responses due to pre-existing immunity limiting LAIV replication [237,238]. The differentiation of Tfh cells, which are critical for germinal center B cell responses, require sustained antigen presentation concurrent with infection-associated signals during the effector phase of 6–8 days post-vaccination [239,240]. LAIV satisfies these dual requirements through prolonged viral replication, whereas IIV fails to provide necessary inflammatory cues, resulting in transient Tfh responses [239]. Moreover, epitope-optimized vaccines in swine broaden CD4+ T cell recognition 4-fold compared to wild-type immunogens, enhancing cross-reactivity to human H1N1pdm09 and avian H5N1 strains through computational design of conserved NP and M1 epitopes [241]. Furthermore, mRNA platforms encoding NP, M1, and PB1 induce bone marrow-resident CD8+ cytotoxic T lymphocytes in ferrets that recognize heterosubtypic H5N1 and H7N9 IAV strains, reducing lung viral loads by 2–3 logs during challenge [242]. Although there is no commercially available vaccine specifically approved for influenza in cattle, a recent study has demonstrated that a replicating RNA (repRNA) vaccine expressing the HA of H5N1 virus isolated from a U.S. dairy cow conferred complete protection against homologous lethal challenge in mice [243]. Therefore, while advanced influenza vaccine platforms, such as LAIV, IIV, and mRNA, demonstrate strong T cell-mediated and cross-protective immunity in humans and animal models [233], no influenza vaccine has yet been approved for use in cattle. However, recent preclinical evidence showing protective efficacy of repRNA-based HA vaccines in murine models highlights the potential for developing T cell-targeted vaccines tailored for bovine hosts [243]. Therefore, the effectiveness of influenza vaccines across species is shaped by both vaccine platform and delivery route, which depends on their ability to induce distinct T cell responses.

## 3. Conclusions

Cattle are important hosts and potential reservoirs for multiple influenza viruses. T cell-mediated immunity plays a central role by supporting antibody production, eliminating infected cells, and providing long-term protection. Although most insights come from human and mouse studies, direct research on bovine T cell responses, particularly those involving TCRαβ+CD4+, TCRαβ+CD8+, and TCRγδ+ T cells, remains scarce, partly due to limited research funding and cattle-specific reagents. Moreover, cattle possess distinct immune features, such as a higher proportion of TCRγδ+ T cells that function as a major regulatory subset, suggesting functional differences from other species. As a result, key gaps remain in understanding the function of bovine T cell subsets during influenza infection, their antigen specificity, tissue-specific features, and the development and maintenance of memory T cells. Addressing these questions will require advanced techniques, such as single-cell RNA sequencing, TCR repertoire profiling, and spatial transcriptomics, combined with functional assays. Cross-species comparisons can also help adapt findings from well-studied models. A better understanding of T cell responses in cattle may facilitate vaccine development, improve animal health, and mitigate the risk of zoonotic influenza transmission and future pandemics.

## Data Availability

No new data was created for this review.
